# Entrance-sealing behavior in the home cage: a defensive response to potential threats linked to the serotonergic system and manifestation of repetitive/stereotypic behavior in mice

**DOI:** 10.3389/fnbeh.2023.1289520

**Published:** 2024-01-05

**Authors:** Noriko Horii-Hayashi, Kazuya Masuda, Taika Kato, Kenta Kobayashi, Ayumu Inutsuka, Miyu F. Nambu, Kazumasa Z. Tanaka, Koichi Inoue, Mayumi Nishi

**Affiliations:** ^1^Anatomy and Cell Biology, Department of Medicine, Nara Medical University, Kashihara, Japan; ^2^Section of Viral Vector Development, National Institute for Physiological Sciences, Okazaki, Japan; ^3^Department of Physiology, Jichi Medical University, Shimono, Japan; ^4^Memory Research Unit, Okinawa Institute of Science and Technology Graduate University (OIST), Kunigami-gun, Japan

**Keywords:** defensive behavior, neuropeptide, hypothalamus, obsessive-compulsive disorder, urocortin-3, anxiety, serotonin, selective serotonin-reuptake inhibitor

## Abstract

The security of animal habitats, such as burrows and nests, is vital for their survival and essential activities, including eating, mating, and raising offspring. Animals instinctively exhibit defensive behaviors to protect themselves from imminent and potential threats. In 1963, researchers reported wild rats sealing the entrances to their burrows from the inside using materials such as mud, sand, and vegetation. This behavior, known as “entrance sealing (ES),” involves repetitive movements of their nose/mouth and forepaws and is likely a proactive measure against potential intruders, which enhances burrow security. These observations provide important insights into the animals’ ability to anticipate potential threats that have not yet occurred and take proactive actions. However, this behavior lacks comprehensive investigation, and the neural mechanisms underpinning it remain unclear. Hypothalamic perifornical neurons expressing urocortin-3 respond to novel objects/potential threats and modulate defensive responses to the objects in mice, including risk assessment and burying. In this study, we further revealed that chemogenetic activation of these neurons elicited ES-like behavior in the home-cage. Furthermore, behavioral changes caused by activating these neurons, including manifestations of ES-like behavior, marble-burying, and risk assessment/burying of a novel object, were effectively suppressed by selective serotonin-reuptake inhibitors. The c-Fos analysis indicated that ES-like behavior was potentially mediated through GABAergic neurons in the lateral septum. These findings underscore the involvement of hypothalamic neurons in the anticipation of potential threats and proactive defense against them. The links of this security system with the manifestation of repetitive/stereotypic behaviors and the serotonergic system provide valuable insights into the mechanisms underlying the symptoms of obsessive-compulsive disorder.

## 1 Introduction

The security of living environments, especially within homes or territories such as nests and burrows, is crucial for animal survival and wellbeing. Secured habitats allow animals to engage in activities essential for their survival and the preservation of species, such as feeding, mating, and rearing offspring. Animals have developed a range of defensive behaviors to protect themselves from threats, including fight/aggression, flight/escape, freezing/immobility, and risk assessment/exploration with vigilance (Blanchard and Blanchard, 2008; Blanchard et al., 2010; Insel et al., 2010; Nicholson and Sommer, 2018; Horii-Hayashi et al., 2021; Smith et al., 2022). Aggression toward intruders, often studied using the resident-intruder paradigm, is one of the defensive behaviors associated with home/territoriality (Koolhaas et al., 2013). Numerous studies utilizing this paradigm have revealed that neurons positive for estrogen receptor α in the ventromedial hypothalamic nucleus (VMH) regulate aggressive behaviors (Lin et al., 2011; Hashikawa et al., 2017; Wang et al., 2019; Zha and Xu, 2021). Interestingly, a study conducted by Calhoun on wild rats reported the existence of another defensive behavior related to home/territory, aimed at defending against potential threats, in addition to aggression (Calhoun, 1963). Calhoun observed and reported that rats often voluntarily seal the entrances to their burrows from the inside using materials like mud, sand, and vegetation (Supplementary Figure 1A; Calhoun, 1963). This behavior, known as “entrance sealing (ES),” involves repetitive movements of their nose and forepaws, as demonstrated in a video available at https://youtu.be/xdgrD1VFx6k (4:16 to 4:54), and serves to create a physical barrier and enhance the security of the burrow, similar to how humans secure their residences to deter intruders. Calhoun also noted that several factors influence the frequency of ES behavior, including lactation, the presence of pups, a lower social rank, and the proximity of traps near the entrances (Calhoun, 1963). These observations provide important insights into the home-related defensive behaviors of wild rats, suggesting that rodents have the abilities to anticipate potential threats and take proactive actions. Nevertheless, similar behavior has not been reported over the past 60 years, and its underlying neural mechanisms remain unclear. Identifying the responsible neurons for the induction of ES behavior is an important issue to understand how animals sense and cope with potential threats. Furthermore, potential threats are closely related to the negative valence of anxiety according to the framework of the Research Domain Criteria (Insel et al., 2010; Nicholson and Sommer, 2018; Smith et al., 2022).

In our mouse breeding facility, and possibly in others, cage lid openings are occasionally observed to be stuffed with bedding material and feces, particularly in cages with lactating females (Supplementary Figure 1B). These observations likely indicate the presence of ES-like behavior in mice. However, it is important to acknowledge that the occurrence of the behavior is uncertain, unpredictable, and variable. Not all lactating females consistently exhibit traces of ES-like behavior, which poses challenges when considering this behavior as a consistent and reliable research subject.

Accumulating evidence supports the diverse physiological and behavioral functions of hypothalamic perifornical (PeF) neurons that express urocortin-3 (UCN3). These functions encompass energy homeostasis, defensive responses to potential threats, and infant-directed attack (Chen et al., 2010, 2012; Kuperman et al., 2010; Autry et al., 2019; Horii-Hayashi et al., 2021). These diverse functions are likely mediated by distinct neuronal populations in specific anatomical locations and projection targets, including the VMH and lateral septum (LS) (Lewis et al., 2001; Li et al., 2002; Kuperman et al., 2010; Autry et al., 2019; Horii-Hayashi et al., 2021). In our recent study, we focused on PeF UCN3 neurons located along the paraventricular nucleus (PVN) in mice (from −0.58 to −0.94 mm to the bregma line) and discovered their involvement in modulating defensive responses to potential threats (Horii-Hayashi et al., 2021). These neurons respond to novel objects/potential threats, but not to acute threats such as predator odor and intruders. Thus, their activity appears to be associated with risk assessment of novel objects, and their activation promotes defensive responses, including risk assessment behavior characterized by sniffing with the stretched attend posture (SAP) in the absence of bedding material and burying in the presence of bedding material (Horii-Hayashi et al., 2021). Additionally, activating these neurons increased marble-burying activity, which is used to construct a common rodent model for assessing repetitive/stereotypic behavior observed in obsessive-compulsive disorder (OCD) (Ichimaru et al., 1995; Deacon, 2006; Taylor et al., 2017; Kraeuter et al., 2019). These findings suggest that PeF UCN3 neurons are involved in defensive responses to potential threats and may be associated with repetitive/stereotypic behavior. However, their specific roles in the home-cage environment and their responses to commonly used pharmacological treatments for anxiety and OCD are not yet fully understood. Further investigation is needed to explore the contribution of PeF UCN3 neurons to defensive behaviors, specifically in the context of the home-cage, and their potential modulation through pharmacological interventions.

In this study, we aimed to elucidate the role of PeF UCN3 neurons in the home-cage context and their potential modulation by anxiolytic and anti-OCD drugs. To selectively activate these neurons, we employed a Gq-based chemogenetic approach using an adeno-associated virus (AAV) vector to express hM3Dq in Ucn3-Cre mice within the PeF region. Subsequently, we observed the resulting behaviors of these mice in the home-cage. Furthermore, we investigated the effects of two clinically used drugs on the observed behaviors within the home-cage, marble-burying activity, and responses to a novel object. The first drug investigated was diazepam (DZP), a commonly prescribed, benzodiazepine-class anxiolytic (Griebel et al., 2000). The second set of drugs consisted of selective serotonin-reuptake inhibitors (SSRIs), including escitalopram (ESC) and fluoxetine (FLX), which are widely utilized for their therapeutic properties in anxiety, OCD, and depression (Jin et al., 2017; Smith et al., 2019). Additionally, we explored the potential involvement of specific projection targets of these neurons, namely the LS and VMH, in eliciting the observed home-cage behaviors. Our present findings support the existence of a home security system against potential threats controlled by hypothalamic neurons. This system is intricately linked to the serotonergic system and the manifestation of repetitive/stereotypic behaviors.

## 2 Materials and methods

### 2.1 Animals drug administration

All animal experimental procedures adhered to the guidelines provided by the Animal Care Committee of Nara Medical University and followed the National Institute of Health Guidelines and the Guidelines for Proper Conduct of Animal Experiments published by the Science Council of Japan. Ucn3-Cre mice were obtained from the Mutant Mouse Resource and Research Center (Stock #: 032078-UCD) and bred as heterozygotes. Male mice aged between 8 and 24 weeks were housed in standard mouse cages with bedding material under standard laboratory conditions (23°C, 55% humidity, and a 12 h light-dark cycle: lights on at 8:00 a.m.). They were provided unrestricted access to both food and water. To ensure unbiased distribution, the mice were age-matched in all experiments and randomly assigned to various experimental groups.

### 2.2 Drug administration

Clozapine N-oxide (CNO) (Abcam, Cambridgeshire, UK) was intraperitoneally administered at a dose of 2 mg/kg. The doses of DZP (Fujifilm Wako Pure Chemical Corporation, Osaka, Japan), ESC (Sigma-Aldrich, St. Louis, MO, USA), and FLX (Tokyo Chemical Industry Co., Ltd., Tokyo, Japan) were selected based on previous studies, at 1 mg/kg (Njung’e and Handley, 1991; Vitale et al., 2008; Jimenez-Gomez et al., 2011; Cueto-Escobedo et al., 2013), 10 mg/kg (Taylor et al., 2017), and 20 mg/kg (Uday et al., 2007; Umathe et al., 2011; Manna and Umathe, 2015), respectively. One of these drugs or a control vehicle was intraperitoneally injected 30 min prior to CNO administration, allowing sufficient time for the drugs to elicit effects before behavioral testing.

### 2.3 Stereotaxic surgery

Stereotaxic surgery was conducted following established procedures (Horii-Hayashi and Nishi, 2021; Horii-Hayashi et al., 2021). AAV vectors [AAV(DJ): -hSyn-FLEX-hM3D(Gq)-mCherry, 1 × 10^13^ copies/mL, Addgene #44361; AAV(DJ): -hSyn-FLEX-mCherry, 1 × 10^13^ copies/mL, Addgene #50459)] (Inutsuka et al., 2014; Horii-Hayashi et al., 2021) were injected into the PeF using the following stereotaxic coordinates: anterior-posterior = −0.82 mm, mid-lateral = ± 0.47 mm, dorsal-ventral = −4.5 mm from the dura matter. The injection volume was 250 nL per hemisphere, delivered at a flow rate of 100 nL/min. Following the surgery, the mice were housed individually for a duration of 4 weeks before being used for the experiments. The accuracy of the injection site was verified through fluorescent microscopic (FluoView 3000; Olympus, Tokyo, Japan) observation in all mice that underwent the surgical procedures.

### 2.4 Immunohistochemistry

The mice were anesthetized with sodium pentobarbital (100 mg/kg) and transcardially perfused with phosphate-buffered saline (PBS), followed by 4% paraformaldehyde in 0.1 M phosphate buffer. Brains were then post-fixed in the same fixative for 16 h at 4°C. Brain sections with a thickness of 50 μm were obtained using a vibratome (Microslicer; Dosaka, Kyoto, Japan). The sections were permeabilized using PBS with 0.3% Triton X-100 (PBST) and blocked with 5% normal horse serum in PBST. Immunofluorescent labeling was performed by incubating the sections with primary antibodies diluted in the blocking solution for 2 days at 4°C. The primary antibodies used were guinea pig anti-c-Fos (dilution, 1:1000; Synaptic System #226004, Gottingen, Germany), rabbit anti-UCN3 (dilution, 1:200; Yanaihara #Y364, Shizuoka, Japan), rabbit anti-ENK (dilution, 1:500; Merck Millipore #AB5024, Burlington, MA, USA), mouse anti-CALB (dilution, 1:1000; Merck Millipore #C9848), and rat anti-mCherry (dilution, 1:200; Thermo Fisher Scientific #M11217, Waltham, MA, USA). After three washes with PBS, the sections were incubated with species-specific secondary antibodies conjugated to Alexa Fluor 405, 488, or 594 (Thermo Fisher Scientific, Osaka, Japan) for 2 h. Following three additional washes with PBS, the sections were mounted on glass slides and sealed using a mounting medium (ProLong Glass Antifade Mountant, Thermo Fisher Scientific # P36981). Fluorescent images were acquired and observed using a confocal microscope (FluoView 3000; Olympus, Tokyo, Japan). To develop immunoreactions by the ABC method using diaminobenzidine (DAB) as the substrate, the ABC kit (Vector laboratories #PK-4000, Newark, CA, USA) and the DAB substrate kit (Vector Laboratories #SK-4100) was utilized following the supplier’s instructions.

### 2.5 Behavioral testing

Behavioral testing was conducted during the light phase of the day, specifically between 9:30 and 13:00. The mice were transferred to a test room at least 20 min before the start of each test. A counterbalanced design was implemented to ensure an unbiased distribution of treatments throughout the experimental sequence. When using the drugs of DZP and SSRIs except for the marble-burying test, the experiments were conducted with a between-group design. In the experiments with a within-subject design, each mouse underwent one test per day, with a minimum 2-day interval between consecutive tests.

#### 2.5.1 Home-cage test

A mouse in the home-cage without a lid was placed in the cage recording apparatus for 20 min to allow it to acclimate to the recording conditions. After the administration of either saline or CNO, home-cage behaviors were recorded and analyzed during the specified time period. ES-like behavior was defined as the behavior in which a mouse pushes and/or piles bedding material toward a wall of the cage using its nose/mouth and forepaws (see Supplementary Movie 2). Rearing was defined as assuming an upright posture on the hind limbs, either with or without the support of a cage wall, while raising the forelimbs off the ground (see Supplementary Movie 3). Grooming was defined as the actions of licking, scratching, nibbling, and rubbing different areas of the body including the face, head, ears, whiskers, and tail (see Supplementary Movie 3). ES-like behavior, rearing, and grooming were assessed by a trained experimenter blinded to the experimental groups. Locomotor activity was analyzed using TopScan LITE software (CleverSys Inc., Reston, VA, USA).

#### 2.5.2 Home-cage test with nesting material

To distinguish between nest-building behavior and ES-like behavior, mice were provided with cotton pads as nesting material 7 days before CNO injection. An experimenter confirmed the presence of nests made with cotton on the day of the test. The procedures for behavioral recording and analysis were carried out in the same way as described in the home-cage test.

#### 2.5.3 New cage test

To assess the influence of the cage conditions on ES-like behavior, mice that exhibited ES-like behavior in their home-cage were transferred to a new cage with fresh bedding material 10 min after CNO administration. Digging was defined as the action in which a mouse moves or removes bedding material using its forepaws and nose/mouth without pushing or piling the material in specified directions (Supplementary Movie 3). Digging, rearing, and grooming were assessed by a trained experimenter blinded to the experimental groups. Locomotor activity was analyzed using TopScan LITE software.

#### 2.5.4 Home-cage test without bedding material

To assess the influence of the absence of bedding material on home-cage behaviors, mice were housed in standard cages with stainless steel wire mesh at the bottom, allowing the collection of feces and urine underneath, for 7 days before the test. The procedures for behavioral recording and analysis were carried out in the same way as described in the home-cage test. Rearing, and grooming were assessed by a trained experimenter who remained blinded to the experimental groups. Locomotor activity was analyzed using TopScan LITE software.

#### 2.5.5 Marble-burying test

The marble-burying test was performed 10 min after the administration of either saline or CNO injection, following established protocols (Deacon, 2006; Thomas et al., 2009). The mice were placed in a standard laboratory cage containing a 4 cm-thick layer of bedding material. On the surface of the bedding, 16 marbles were evenly arranged in a 4 × 4 grid pattern. During the 30 min test, the mice were allowed to move freely within the cage. Marbles were considered buried when they were covered by at least two-thirds of the bedding material.

#### 2.5.6 Novel object-burying test

The novel object-burying test was conducted using the procedures outlined in our previous studies (Horii-Hayashi and Nishi, 2021; Horii-Hayashi et al., 2021). Sniffing behavior was defined as instances where the distance between the mouse’s nose and the object was < 1 cm. Burying behavior was defined as the action in which a mouse pushes bedding material toward an object, either with or without covering the object. Burying behavior and sniffing behavior accompanied with the SAP were assessed by a trained experimenter who remained blinded to the experimental groups. Locomotor activity was analyzed using TopScan LITE.

### 2.6 Corticosterone/adrenaline assay

Mice were injected with saline or CNO (2 mg/kg). Thirty minutes after injection, blood samples were collected from the facial vein with an animal lancet. Blood samples were centrifuged at 2,000 × *g* for 15 min at 4°C, and plasma was collected and stored at −85°C. Corticosterone concentration was measured using an enzyme immunoassay kit (Yanaihara Institute #YK240, Fujinomiya, Japan), and adrenaline concentration was measured using an epinephrine enzyme-linked immunosorbent assay (ELISA) kit (Abnova corporation #KA1882, Taipei, Taiwan).

### 2.7 Statistical analysis

Statistical analyses were performed using GraphPad Prism 9 (GraphPad Software, San Diego, CA, USA). The normality of the distribution was assessed using the Shapiro-Wilk test. When a normal distribution was confirmed, two-group comparisons were carried out using either paired or unpaired *t*-tests. For comparisons involving three or more groups, one-way ANOVA with Tukey’s *post-hoc* multiple comparison test was performed. If normality was not observed, the Wilcoxon signed-rank test was used for two-group comparisons with paired data, and the Kruskal-Wallis’s test was employed for comparisons involving three or more groups, followed by the Dunnett’s *post-hoc* test for multiple comparisons. A significance level of *P* < 0.05 was used to indicate statistical significance.

### 2.8 Graphs and illustrations

Graphs were created using GraphPad Prism 9 software. Illustrations were created using the online tool BioRender.

## 3 Results

### 3.1 Chemogenetic activation of PeF UCN3 neurons

To selectively activate PeF UCN3 neurons, we utilized AAV injection techniques, which allowed for Cre-dependent expression of either hM3Dq-mCherry or mCherry alone as a control in Ucn3-Cre mice (Supplementary Figure 2A). In the brain sections of mice undergoing surgery, we observed mCherry-labeled cells in the PeF from −0.58 mm to −0.94 mm to the bregma line (Supplementary Figure 2B), replicating previous results (Horii-Hayashi et al., 2021). UCN3 immunolabeling revealed the co-localization of mCherry-labeled cells and UCN3-labeled cells (Supplementary Figure 2C). To confirm the activation of these neurons by CNO administration, we performed immunolabeling of c-Fos, a marker for activated neurons, in saline- or CNO-injected mice. The results demonstrated that CNO administration induced c-Fos expression in 96.0 ± 0.34% of mCherry-labeled cells, while saline injection resulted in c-Fos expression in only 2.12 ± 0.47% of mCherry-positive cells (Supplementary Figures 2D, E) (*n* = 5. 5; unpaired *t*-test, *t* (9) = 34.35, ****P < 0.0001). This confirmed the activation of PeF UCN3 neurons by CNO administration.

### 3.2 CNO administration induced ES-like behavior in the home-cage

**FIGURE 1:**
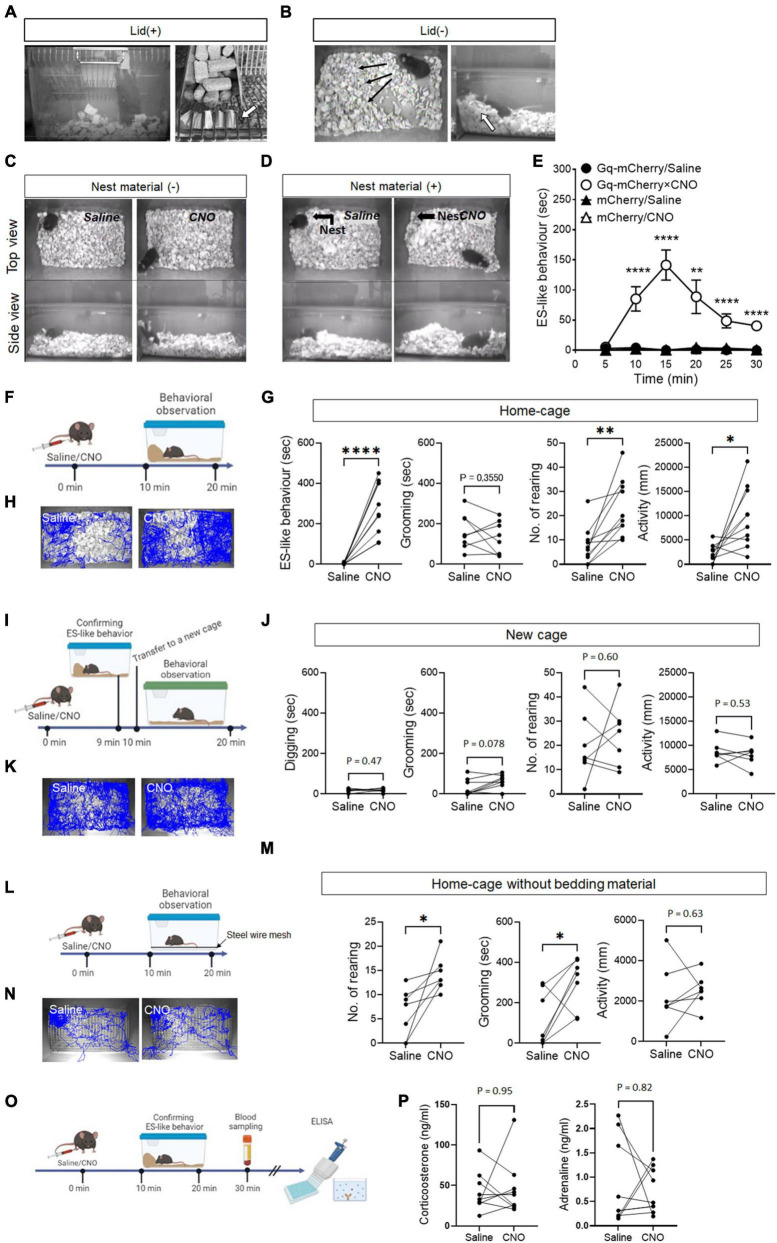
ES-like behavior induced by the activation of PeF UCN3 neurons **(A,B)** Pictures showing ES-like behavior observed after CNO injection when the cage lid is present **(A)**, with an arrow highlighting bedding material sealing the lid openings, and when the lid is absent **(B)**, with an arrow indicating a pile of bedding material along the cage wall. **(C,D)** Pictures showing ES-like behavior observed after CNO injection in the absence **(C)** and presence **(D)** of nest material. **(E)** Time-course changes in ES-like behavior following saline or CNO injection in mice subjected to viral injection of Gq-mCherry or mCherry vector (*n* = 6, Turkey’s *post-hoc* results of Gq-mCherry/Saline vs. Gq-mCherry/CNO following unpaired one-way ANOVA are indicated in the graph). **(F)** Timeline of behavioral observation within the home-cage following saline or CNO injection (Time 0 min: saline/CNO administration, Time 10–20 min: behavioral observation within the home-cage). **(G)** Graphs showing the duration of ES-like behavior, number of rearing, duration of grooming behavior, and activity in the home-cage following saline or CNO injection (*n* = 10, the results of paired *t*-test are indicated). **(H)** Tracking of the mice body center injected with saline (left) or CNO (right) in the home-cage. **(I)** Timeline of behavioral observation in a new cage following saline or CNO injection (Time 0 min: saline/CNO administration, Time 9 min: Observation and confirmation of the incidence of ES-like behavior, Time 10–20 min: behavioral observation within a new cage). **(J)** Graphs showing the duration of digging behavior, the number of rearing, the duration of grooming behavior, and activity in a new cage following saline or CNO (*n* = 7, Digging, No. of rearing, and activity were analyzed by paired *t*-test. Grooming was done by Wilcoxon-signed rank test). **(K)** Tracking of the body center of mice injected with saline (left) or CNO (right) in a new cage. **(L)** Home-cage test without bedding material **(M)** Graphs showing the number of rearing, the duration of grooming behavior, and activity in the home-cage without bedding material following saline or CNO (*n* = 7, the results of paired *t*-test are indicated). **(N)** Tracking of the body center of mice injected with saline (left) or CNO (right). **(O)** Timeline of blood sampling after ES-like behavior induction by CNO administration (Time 0 min: CNO administration, Time 10–20 min: Observation and confirmation of the incidence of ES-like behavior, Time 30 min: Blood sampling from mice). **(P)** Plasma corticosterone and adrenaline levels were quantified using ELISA techniques. (*n* = 9, The results of Wilcoxon signed-rank test are indicated. Corticosterone: saline, 42.07 ± 8.05 ng/mL, CNO, 46.22 ± 11.53 ng/mL; Adrenaline: saline 0.87 ± 0.29 ng/mL, CNO 0.72 ± 0.15 ng/mL). *P < 0.05, **P < 0.01, ****P < 0.001.

In a home-cage equipped with a grid lid, CNO-injected mice exhibited distinct behaviors, characterized by holding bedding material in their mouth and/or forepaws, rearing up, and plugging the openings of the grid lid (Figure 1A; Supplementary Movie 1). In a home-cage without a grid lid, the mice exhibited behaviors such as pushing and piling bedding material in a corner of the cage, as well as rearing up while holding bedding material in their mouth and/or forepaws (Figure 1B; Supplementary Movie 2). The behavioral characteristics induced by CNO exhibited similarities to ES behavior observed in rats (Calhoun, 1963), which specifically include: (i) the subjects plugging the openings leading to the outside of their habitats using materials, (ii) the behaviors taking place from inside their respective habitats, and (iii) the behaviors being accomplished through repetitive movements of the nose/mouth and forepaws. Based on these similarities, we refer to the behavior induced by CNO within the home-cage as “ES-like behavior” (Supplementary Movie 2).

To investigate whether ES-like behavior is distinct from nesting behavior, we conducted an experiment where mice were provided with cotton pads as nest material for 7 days before CNO injection (Figure 1D). On the day of CNO injection, we observed and confirmed the construction of cotton-made nests (Figure 1D). However, CNO-injected mice did not interact with the cotton-made nest and exhibited behaviors similar to those observed in the absence of cotton pads (Figures 1C, D; Supplementary Movie 4). These results indicate that the behavior induced by CNO is distinct from nesting behavior.

Time-course changes in ES-like behavior were examined in the home-cage without the cage lid using mice injected with saline or CNO and those who had received viral injection of hM3Dq(Gq)-mCherry or mCherry (control) (Figure 1E). Significant differences were observed among the 4 groups from 10 min to 30 min after CNO injection (Figure 1E) [*n* = 6, one-way ANOVA, 0–5 min: *F*(3,20) = 2.08, *P* = 0.14; 5–10 min: *F*(3,20) = 16.35, ****P < 0.0001; 10–15 min: *F*(3,20) = 32.30, ****P < 0.0001; 15–20 min: *F*(3,20) = 9.669, ***P < 0.001; 20–15 min, *F*(3,20) = 15.86, ****P < 0.0001; 25–30 min: *F*(3,20) = 45.39, ****P < 0.0001]. ES-like behavior was prominently observed in mice that had received viral injection of Gq-mCherry approximately 10 min after CNO administration, whereas the behavior was not observed after saline injection to the mice that had received the same viral injection (Figure 1E) (*n* = 6, Turkey’s *post-hoc* test, Gq-mCherry/saline vs. Gq-mCherry/CNO, 0–5 min, *P* = 0.79; 5–10 min: ****P < 0.0001; 10–15 min: ****P < 0.0001; 15–20 min: ** *P* < 0.01; 20–25 min: ****P < 0.0001; 25–30 min: ****P < 0.0001). The behavior reached its peak at 15 min after CNO injection and persisted for an additional 15 min (Figure 1E). Mice that received either saline or CNO injection following viral injection of the mCherry control vector did not display ES-like behavior (Figure 1E). These results indicate that ES-like behavior was not an artifactual behavior induced by CNO administration.

In addition to analyzing ES-like behavior, we examined other typical behaviors within the home-cage, including rearing, grooming (see Methods and Supplementary Movie 3), and locomotion, 10–20 min after CNO administration (Figure 1F). The results showed that CNO administration significantly increased the frequency of rearing and locomotor activity compared with those with the saline injection (Figures 1G, H) [*n* = 10, paired *t*-test, ES-like behavior: *t* (9) = 6.76, ****P < 0.0001; No. of rearing: *t* (9) = 4.28, **P < 0.01; locomotor activity: *t* (9) = 3.05, *P < 0.05]. However, CNO administration did not affect the duration of grooming [*n* = 10, paired *t*-test, *t* (9) = 0.9903, *P* = 0.36].

To further examine whether ES-like behavior can be elicited in a new cage, we performed an experiment involving the transfer of mice exhibiting ES-like behavior to a new cage containing fresh bedding material (Figure 1I). However, the mice in the new cage did not display any specific behavior indicative of ES. Therefore, we focused on measuring digging behavior observable in the cage, which was defined as the action of moving or removing bedding material using the forepaws and nose/mouth without pushing or piling the material in specified directions (Supplementary Movie 3) in addition to rearing, grooming, and locomotor activity. Nevertheless, we did not observe any significant differences in the measured behaviors between the saline and CNO groups (Figures 1J, K) [*n* = 7, paired *t*-test, Digging: *t* (6) = 0.77, *P* = 0.47; No. of rearing: *t* (6) = 0.55, *P* = 0.60; Activity: *t* (6) = 0.65, *P* = 0.54; Wilcoxon signed-rank test, Grooming: *P* = 0.078]. These results suggest that the presence of bedding material alone is not enough to induce ES-like behavior in mice. Instead, the context of the home-cage plays a crucial role in eliciting this behavior. This finding supports the notion that the observed behavior is associated with the home-cage environment, similar to ES observed within rat burrows (Calhoun, 1963).

The influence of the absence of bedding material on ES-like behavior was investigated. Mice were housed in standard cages with stainless steel wire mesh at the bottom, allowing the collection of feces and urine underneath, for 7 days before to the test (Figure 1L). CNO administration significantly increased the frequency of rearing [*n* = 7, paired *t*-test, *t* (6) = 3.10, *P < 0.05] and the duration of grooming [*t* (6) = 2.60, *P > 0.05] without affecting locomotor activity [*t* (6) = 0.60, *P* = 0.63] (Figures 1M, N). These results suggest that grooming can serve as a substitute behavior for ES-like behavior when the intended behavior cannot be performed due to the unavailability of suitable material.

### 3.3 Lack of effects of CNO administration on plasma corticosterone and adrenaline levels

Defensive responses caused by imminent threats, such as the fight-or-flight responses, are associated with the activation of the sympathetic nervous system (Cannon, 1915; Jansen et al., 1995). Stressful events, such as receiving electric shocks and experiencing acute or chronic restraint stress, increase the plasma corticosterone levels in mice (Shanks et al., 1990; Sadler and Bailey, 2016). To investigate whether ES-like behavior is associated with changes in stress hormone levels and the activation of the sympathetic nervous system, plasma samples were collected from mice 30 min after CNO injection, specifically from mice that had exhibited ES-like behavior (Figure 1O). However, we did not observe significant differences in corticosterone and adrenaline levels between the saline and CNO groups (Figure 1P) (*n* = 9, Wilcoxon signed-rank test, corticosterone: saline, 42.07 ± 8.05 ng/mL, CNO, 46.22 ± 11.53 ng/mL, *P* = 0.95; adrenaline: saline 0.87 ± 0.29 ng/mL, CNO 0.72 ± 0.15 ng/mL, *P* = 0.82). These results suggest that the induction of ES-like behavior occurred without changes in stress hormone and adrenalin levels.

### 3.4 Suppression of ES-like behavior induced by CNO through DZP and SSRIs

**FIGURE 2:**
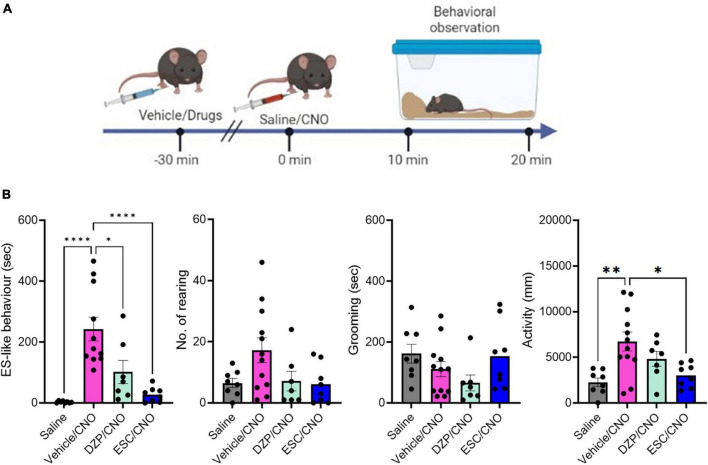
The effects of DZP and ESC on home-cage behaviors. **(A)** Timeline of behavioral observation in mice after injection with vehicle or drugs, followed by CNO administration (Time –30 min: injection of vehicle or drugs, Time 0 min: CNO administration, Time 10–20 min: behavioral observation within the home-cage). **(B)** Graphs showing the duration of ES-like behavior, the number of rearing, the duration of grooming behavior, and activity (*n* = 8–11, ES-like behavior, No. of rearing, and activity are analyzed by one-way ANOVA followed by Turkey’s *post-hoc* test. Grooming is analyzed by Kruskal-Wallis’s test). *P < 0.05, **P < 0.001, ****P < 0.0001.

To examine the effects of DZP and SSRIs on ES-like behavior, we treated mice with DZP at a dose of 1 mg/kg (*n* = 8), ESC at a dose of 10 mg/kg (*n* = 8), FLX at a dose of 20 mg/kg (*n* = 8), or vehicle/control (*n* = 11) 30 min before CNO injection (Figure 2A). Significant differences were observed in ES-like behavior [*n* = 8–11, one-way ANOVA, *F*(3,31) = 14.74, ****P < 0.0001] and activity [*F*(3,31) = 6.25, **P < 0.01] among the 4 groups, but not in the number of rearing [*F*(3,31) = 2.784, *P* = 0.0578] and the time spent on grooming (Kruskal-Wallis, *P* = 0.10).

The group treated with vehicle (Vehicle/CNO) showed significant increases in ES-like behavior (Turkey’s *post-hoc* test, ****P < 0.0001) and activity (Turkey’s *post-hoc* test, **P < 0.01) (Figure 2B). Treatment with ESC (ESC/CNO) significantly reduced both ES-like behavior (Turkey’s *post-hoc* test, ****P < 0.0001) and activity (Turkey’s *post-hoc* test, *P < 0.05), whereas treatment with DZP (DZP/CNO) significantly reduced ES-like behavior (Turkey’s *post-hoc* test, *P < 0.05), compared with those in the vehicle-treated group (Vehicle/CNO) (Figure 2B). Additionally, the effects in the FLX-treated group were similar to those in the ESC-treated group (Supplementary Figure 3; Supplementary Movie 5). Significant differences were observed in ES-like behavior [one-way ANOVA, *F*(3,30) = 12.55, ****P < 0.0001] and activity [one-way ANOVA, *F*(3,30) = 14.74, ****P < 0.0001]. Compared with the vehicle-treated group, the FLX-treated group (FLX/CNO) showed significant reductions in both ES-like behavior (Turkey’s *post-hoc* test, ***P < 0.001) and activity (Turkey’s *post-hoc* test, *P < 0.01). These results suggest that although both DZP and SSRIs decrease ES-like behavior induced by the activation of PeF UCN3 neurons, SSRIs are more effective to suppress the behavior.

### 3.5 Suppression of CNO-induced increase in marble-burying activity by SSRIs

**FIGURE 3:**
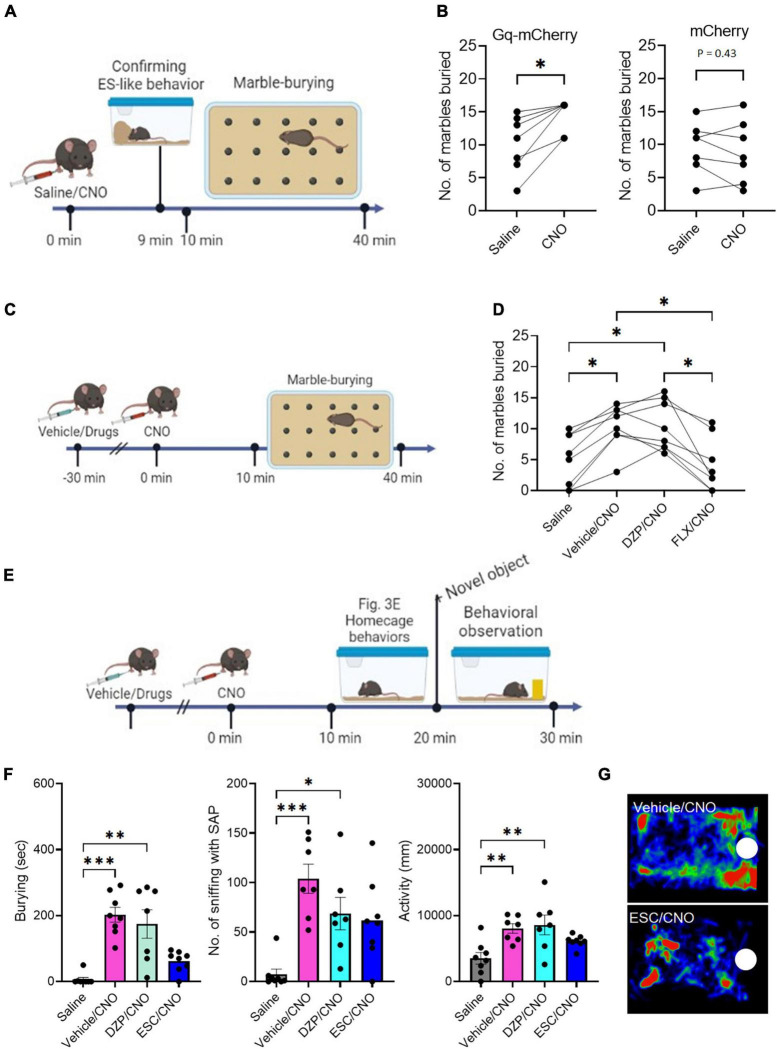
The effects of DZP and ESC on burying behavior **(A)** Timeline of marble-burying test after CNO administration (Time 0 min: saline/CNO administration, Time 9 min: Confirmation of the incidence of ES-like behavior, Time 10–40 min: marble-burying test). **(B)** Graphs showing the number of marbles buried after saline or CNO administration in mice that received viral injection of Gq-mCherry vector (left) or mCherry vector (right) (Gq-mCherry: *n* = 7, Wilcoxon signed-rank test; mCherry: Paired *t*-test). **(C)** Timeline of marble-burying test in mice after injection with vehicle or drugs, followed by CNO administration (Time –30 min: injection of vehicle or drugs, Time 0 min: CNO administration, Time 10–40 min: marble-burying test). **(D)** Graph showing the number of marbles buried in mice after injection with vehicle, DZP, or FLX, followed by CNO administration (*n* = 8, repeated one-way ANOVA followed by Turkey’s *post-hoc* test). **(E)** Timeline of object-burying test in the home-cage after injection with vehicle or drugs, followed by CNO administration (Time –30 min: injection of vehicle or drugs, Time 0 min: saline/CNO administration, Time 10–20: Observation of home-cage behavior, Time 20–30 min: observation of behavioral responses to a novel object). **(F)** Graphs showing the duration of burying, number of sniffing with SAP, duration of sniffing, and activity in mice after injection of vehicle, DZP, or ESC, followed by CNO administration (*n* = 7–8, Burying and No. of sniffing with SAP are analyzed by Kruskal-Wallis’s test followed by Dunnett’s *post-hoc* test. Activity is analyzed by one-way ANOVA followed by Turkey’s *post-hoc* test). **(G)** Representative images of the heat maps showing the stay time of mice treated with vehicle (top) and ESC (bottom). Maps are generated by tracking the movement of the mouse’s nose over a specific period of time. The color intensity on the map represents the amount of time the mouse spent in each location, with warmer colors indicating longer stay times. White circles indicate the location of a novel object. *P < 0.05, **P < 0.01, ***P < 0.001.

To assess whether ES-like behavior is associated with burying activity, we conducted the marble-burying test using mice engaged in ES-like behavior within the home-cage (Figure 3A). The number of marbles buried by mice that exhibited ES-like behavior was higher than that by saline-injected mice (Figure 3B) (*n* = 7, Wilcoxon signed-rank test, *P < 0.05). However, there was no significant difference in the number of buried marbles between the saline and CNO groups among mice that received viral injection of the mCherry control vector (Figure 3B) [*n* = 7, paired *t*-test, *t* (6) = 0.85, *P* = 0.43].

To investigate the effects of DZP and FLX on the increased marble-burying activity induced by CNO administration, mice were treated with the vehicle, DZP, or FLX 30 min before CNO administration (Figure 3C). There was a significant difference among the 4 groups [*n* = 8, one-way ANOVA, *F*(2.270,15.89) = 15.08, ****P < 0.0001]. Treatment with the vehicle (Vehicle/CNO) or DZP (DZP/CNO) significantly increased marble-burying activity compared to saline injection (Figure 3D) (Turkey’s *post-hoc* test, saline vs. vehicle/CNO, *P < 0.05; saline vs. DZP/CNO, *P < 0.05). However, treatment with FLX (FLX/CNO) significantly reduced the activity compared to vehicle treatment (Turkey’s *post-hoc* test, *P < 0.05) and DZP treatment (Turkey’s *post-hoc* test, *P < 0.05) (Figure 3D). The FLX treatment reduced the activity to a level similar to saline injection (Turkey’s *post-hoc* test, *P* = 0.93). These results suggest that the induction mechanism of ES-like behavior overlaps with the enhanced mechanism of marble-burying activity and both are responsive to SSRIs.

### 3.6 Suppression of CNO-induced increase in responses to a novel object by SSRIs

Previously, we demonstrated that the activation of PeF UCN3 neurons increased sniffing with the SAP toward a novel object, which serves as a behavioral marker for risk assessment, as well as burying behavior (Horii-Hayashi et al., 2021). In the present study, we further examined the effects of DZP, ESC, and FLX on these enhanced responses to a novel object placed in the home-cage (Figure 3E). Significant differences were observed in burying (*n* = 7–8, Kruskal-Wallis’s test, ****P < 0.0001), the number of sniffing with SAP (*n* = 7–8, Kruskal-Wallis’s test, **P < 0.01), and activity [*n* = 7–8, one-way ANOVA, *F*(3,26) = 6.23, **P < 0.01]. Compared with the saline-injected group, the group injected with vehicle followed by CNO (vehicle/CNO) showed significant increases in burying (Dunnett’s *post-hoc* test, ***P < 0.001), the number of sniffing with the SAP (Dunnett’s *post-hoc* test, ***P < 0.001), and activity (Turkey’s *post-hoc* test, **P < 0.014), indicating heightened defensive responses to a potential threat (Figure 3F). Compared with the saline-injected group, the group treated with DZP followed by CNO (DZP/CNO) exhibited significant increases in burying (Dunnett’s *post-hoc* test, **P < 0.01), the number of sniffing with the SAP (Dunnett’s *post-hoc* test, **P < 0.01), and activity (Turkey’s *post-hoc* test, **P < 0.01) (Figure 3F). However, the group treated with ESC followed by CNO (ESC/CNO) exhibited similar levels of burying, sniffing with SAP, and activity to the saline-injected group (Figures 3F, G) (Burying: Dunnett’s *post-hoc* test, *P* = 0.53; No, of sniffing with the SAP, Dunnett’s *post-hoc* test, *P* = 0.12; Activity, Turkey’s *post-hoc* test, *P* = 0.53). Treatment with FLX showed similar effects to ESC (Supplementary Figure 4; Supplementary Movie 6). There were significant differences among the 4 groups in burying (Kruskal-Wallis’s test, ***P < 0.001), the number of sniffing with SAP (Kruskal-Wallis’s test, ***P < 0.001), and activity [one-way ANOVA, *F*(3,26) = 5.463, **P < 0.01]. Treatment with FLX (FLX/CNO) suppressed burying, the number of sniffing with SAP, and activity at levels similar to those in the saline-injected group (Burying, Dunnett’s *post-hoc* test, ****P > 0.99; No. of sniffing with SAP, Dunnett’s *post-hoc* test, *P* = 0.35; Activity, Turkey’s *post-hoc* test, *P* = 0.68). These results suggest that SSRIs suppress the heightened responses to potential threats induced by the activation of PeF UCN3 neurons within the home-cage.

### 3.7 The c-Fos expression of projection targets of PeF UCN3 neurons after the induction of ES-like behavior

**FIGURE 4:**
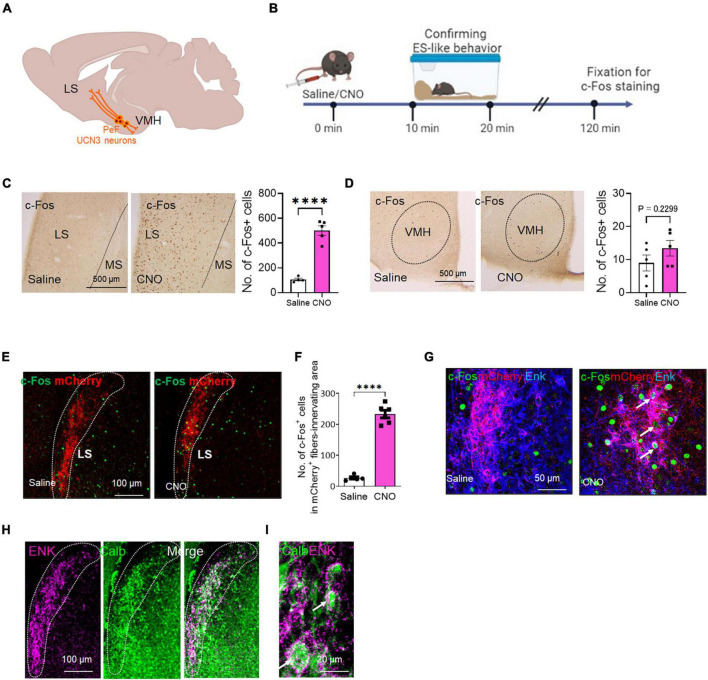
The c-Fos expression of the projection targets of PeF UCN3 neurons following the induction of ES-like behavior **(A)** Schematic diagram illustrating the projection targets of PeF UCN3 neurons. **(B)** Timeline of c-Fos expression study after the induction of ES-like behavior (Time 0 min: saline/CNO administration, Time 10–20 min: observation and confirmation of ES-like behavior, Time 120 min: fixation for immunohistochemical staining). **(C,D)** Pictures showing c-Fos-labeled cells in the LS **(C)** and VMH **(D)** of mice injected with saline (left) or CNO (right). Graphs showing the number of c-Fos-labeled cells (*n* = 5, unpaired *t*-test. LS: saline 105 ± 10.77; CNO 500 ± 37.8; VMH: saline 9.0 ± 2.4; CNO 13.4 ± 2.4). **(E)** Lower resolution images of fluorescent double labeling of c-Fos (green) and mCherry (red) in the LS of mice injected with saline (left) or CNO (right). Dotted lines delineate regions innervated by mCherry-labeled fibers. **(F)** Graph showing the number of c-Fos-labeled cells in the regions innervated with mCherry-labeled fibers (*n* = 5–6, unpaired *t*-test, saline: 27.0 ± 8.6, CNO: 232 ± 30.2). **(G)** Higher resolution images of triple labeling of c-Fos (green), mCherry (red), and Enk (blue) in the LS of mice injected with saline (left) or CNO (right). Arrows indicates c-Fos-labeled cells surrounded by mCherry and Enk double-labeled nerve fibers. **(H,I)** Lower **(H)** and higher **(I)** resolution images of double labeling of ENK (magenta) and CALB (green) in the LS. Doted lines delineate the regions innervated with ENK-labeled fibers. Arrows in **(I)** indicate CALB-labeled cells surrounded by ENK-labeled nerve fibers. ****P < 0.001.

PeF UCN3 neurons project to both the LS and VMH (Lewis et al., 2001; Li et al., 2002; Kuperman et al., 2010; Horii-Hayashi et al., 2021; Figure 4A). Notably, PeF UCN3 neurons projecting to the LS co-express enkephalin (ENK) and form perisomatic baskets around LS neurons (Chen et al., 2011; Horii-Hayashi et al., 2021). We also confirmed the overlap of UCN3-labeled nerve fibers with ENK-labeled fibers in the LS, particularly in the posterior part (at 0.62 to 0.38 mm to the bregma line) (Supplementary Figure 5A). However, in the VMH, UCN3-labeled fibers were observed without such overlapping (Supplementary Figure 5B). To investigate the involvement of these projection targets in ES-like behavior, we analyzed c-Fos expression in the posterior part of the LS and VMH following the induction of ES-like behavior by CNO (Figure 4B). The results indicated a significant increase in the number of c-Fos-labeled cells in the LS (Figure 4C) [*n* = 5, saline 105 ± 10.77, CNO 500 ± 37.8; unpaired *t*-test, *t* (7) = 9.0, ****P < 0.0001], but not in the VMH (Figure 4D) [*n* = 5, saline 9.0 ± 2.4, CNO 13.4 ± 2.4; unpaired *t*-test, *t* (8) = 1.30, *P* = 0.23]. To further investigate whether PeF UCN3 neurons directly activate their target neurons, we performed immunofluorescent labeling of mCherry and ENK coupled with c-Fos. The results revealed that c-Fos expression was observed within the region innervated by mCherry-expressing nerve fibers, particularly in mice injected with CNO, but not with saline (Figure 4E). The number of c-Fos-labeled cells in the region identified by the innervation of mCherry-labeled fibers was significantly higher in CNO-injected mice than in saline-injected mice (Figure 4F) [*n* = 5–6, saline: 27.0 ± 8.6, CNO: 232 ± 30.2; unpaired *t*-test, *t* (9) = 14.62, ****P < 0.0001, **P < 0.01]. Higher resolution images demonstrated that mCherry/Enk-labeled nerve fibers formed perisomatic baskets (Figure 4G) and, importantly, the c-Fos signal was observed in neurons surrounded by the baskets in CNO-injected mice but not in saline-injected mice (Figure 4G). These results suggest that PeF UCN3 neurons directly activate their target neurons.

A previous study in mice has demonstrated that over 90% of LS neurons are γ-aminobutyric acid (GABA)ergic and express interneuron markers calbindin (CALB) or calretinin (Zhao et al., 2013). We next identified the subtypes of LS neurons innervated by PeF UCN3/ENK neurons. The results indicated that the majority of ENK-labeled nerve fibers surrounded CALB-labeled neurons (Figures 4H, I). These findings suggest that ES-like behavior may be mediated through the LS, specifically involving a population of CALB-labeled GABAergic neurons.

## 4 Discussion

**FIGURE 5:**
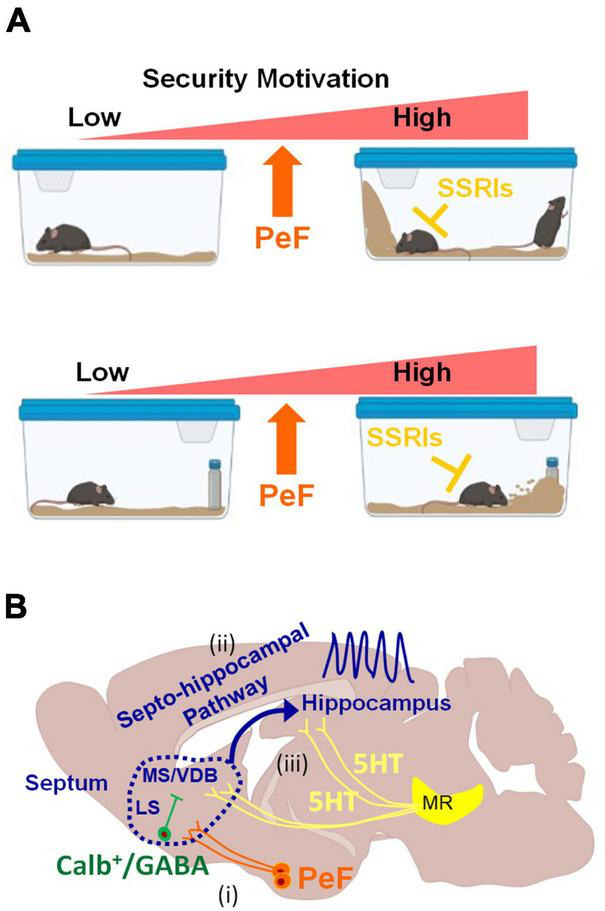
Schematic diagrams illustrating behaviors induced by the activation of PeF UCN3 neurons and potential downstream neural circuits **(A)** Heightened motivation for home-cage security through the activation of PeF UCN3 neurons: the activation of these neurons forms a barrier by ES-like behavior, increases rearing to check potential threats in the home-cage without object (top). The same manipulation also increases checking/risk assessment and burying of a novel object as coping behaviors toward a potential threat (bottom). ES-like behavior, checking/risk assessment, and burying can be efficiently suppressed by SSRIs. **(B)** Potential downstream neural circuits of PeF UCN3 neurons providing a plausible explanation for the observed phenomena in this study: (i) PeF UCN3 neurons innervate to CALB-expressing GABAergic neurons in the LS, which relay the signals to their downstream of the MS/VDB complex (Leranth and Frotscher, 1989). (ii) The MS/VDB complex is connected to the hippocampus through the septo-hippocampal pathway, which generates hippocampal theta oscillation (Vertes and Kocsis, 1997) and promotes exploration of novel objects (Gangadharan et al., 2016). (iii) The theta rhythm can be desynchronized by serotonergic inputs from the median raphe (MR) (Graeff et al., 1980; Jelitai et al., 2021).

A secure habitat is crucial for animals to fulfill their basic needs and ensure successful reproduction (Calhoun, 1963). A previous study has identified a distinct burrow security system in wild rats, operating independently from a defensive system against immediate threats. In our study, activation of PeF UCN3 neurons in mice induced ES-like behavior and increased rearing within the home-cage (Figure 5A) without altering corticosterone and adrenaline levels. Subsequently, SSRIs efficiently suppressed the behaviors modulated by PeF UCN3 neuron activation, including ES-like behavior, marble-burying, risk assessment, and burying of a novel object, suggesting that downstream circuits are potentially regulated by the serotonergic system. The commonality between ES-like behavior and risk assessment/burying of a novel object is their anticipation of potential threats that have not yet occurred and proactive defensiveness against them. Ensuring safety within their home territory is crucial for animals. Even in the absence of actual potential threat stimuli like novel objects, it is likely that animals are innately programmed to exhibit defensive behaviors that anticipate potential intruders. Analysis of c-Fos expression and histological examination indicated the likely role of the LS, rather than the VMH, as the mediator of these behaviors. Specifically, we discovered a distinct population of CALB-expressing GABAergic neurons within the LS that may play a significant role in these behaviors. This finding is supported by the results of previous studies demonstrating a marked reduction in burying behavior following the infusion of midazolam or muscimol into the LS (Pesold and Treit, 1996; Degroot et al., 2001). Based on our findings and previous research, we propose that PeF UCN3 neurons play a role in enhancing the security of the home environment by activating LS GABAergic neurons, potentially regulated by the serotonergic system, and contribute to the manifestation of repetitive/stereotypic behaviors. However, further investigations are needed to identify the neural pathways eliciting ES-like behavior through an optogenetic approach targeting individual specific pathways.

The septo-hippocampal pathway is proposed as a potential downstream circuit of PeF UCN3 neurons, explaining all observed phenomena in this study (Figure 5B). This pathway connects the medial septum (MS)/vertical limb of the diagonal band of Broca (VDB) complex to the hippocampus, playing a crucial role in cognitive processes and behaviors (Müller and Remy, 2018). It delivers cholinergic and GABAergic projections from the MS/VDB to the hippocampus, contributing to hippocampal theta oscillations. These oscillations are associated with locomotion, attention, object exploration, and spatial memory and learning (Dutar et al., 1995; Fuhrmann et al., 2015; Mamad et al., 2015; Gangadharan et al., 2016; Tsanov, 2017; Solórzano Hernández et al., 2022). Based on the accumulating evidence, we hypothesize its involvement in ES-like behavior and defensive responses to a novel object (Figure 5B). First, this pathway promotes exploration of a novel object through hippocampal theta oscillations (Gangadharan et al., 2016). Second, muscimol infusion into the MS or LS reduces burying behavior (Degroot et al., 2001). Third, hippocampal lesions and physostigmine (a cholinesterase inhibitor) infusion decrease burying behavior (Degroot and Treit, 2002; Deacon, 2006). Fourth, GABAergic neurons in the LS project to MS cholinergic neurons (Leranth and Frotscher, 1989). Lastly, serotoninergic inputs from the median raphe desynchronize theta oscillations (Freund, 1992; Vertes and Kocsis, 1997; Gutiérrez-Guzmán et al., 2017; Solórzano Hernández et al., 2022). These suggest the potential involvement of this pathway in burying behavior and exploring novel objects under the control of the serotonergic system. Further investigations are required to elucidate the precise mechanisms underlying the contributions of this pathway to the observed behaviors.

Marble-burying is widely used to assess repetitive/stereotypic behavior in rodents (Ichimaru et al., 1995; Deacon, 2006; Wolmarans et al., 2016; Taylor et al., 2017). OCD is characterized by obsessive thoughts and compulsive behaviors, such as repetitive checking and excessive handwashing (Robbins et al., 2019). Individuals with OCD exhibit cognitive biases related to perceiving potential threats and difficulty tolerating uncertainty (Sookman and Pinard, 2002; Gentes and Ruscio, 2011; Pinciotti et al., 2021). SSRIs are considered the most effective treatment for OCD (Bloch and Storch, 2015; Hirschtritt et al., 2017; Goodman et al., 2021), while benzodiazepines are ineffective (Robbins et al., 2019). The activation of PeF UCN3 neurons in this study induced repetitive/stereotypic behaviors resembling OCD, and SSRIs inhibited all behaviors induced by CNO administration at the same levels as saline injection. One potential mechanism underlying SSRI efficacy involves influencing the downstream circuit of PeF UCN3 neurons, including the LS and its subsequent pathways. Another mechanism is the direct impact of serotonin on PeF UCN3 neurons. Both mechanisms are supported by previous histological studies investigating the distributions of serotonergic fibers in rodents (Steinbusch, 1981; Awasthi et al., 2021), which indicate abundant serotonergic innervations in the PeF, septum, and components of the cortico-striato-thalamo-cortical (CSTC) loops strongly implicated in the pathophysiology of OCD.

In this study, acute administration was sufficient to inhibit repetitive/stereotypic behaviors, whereas chronic administration is typically required for therapeutic effects in OCD (Szechtman and Woody, 2006; Woody and Szechtman, 2011; Woody et al., 2019). This difference may be attributed to the underlying brain regions that are dysfunctional in OCD but were not directly manipulated in this study. Another possibility is that CNO administration does not mimic the circuitry of OCD, because it represents an acute manipulation. The CSTC loops comprise the sensorimotor, associative, and limbic loops, and play a significant role in regulating motor, cognitive, and emotional processes, respectively (Pauls et al., 2014; Gonçalves et al., 2016; Monteiro and Feng, 2016; Gao et al., 2019; Goodman et al., 2021). Among these loops, the limbic loop, which encompasses the paralimbic cortex and limbic structures such as the hippocampus and amygdala, has been specifically implicated in the pathophysiology of OCD (Gonçalves et al., 2016; Boedhoe et al., 2017; Rao et al., 2018; Gurok et al., 2019). Furthermore, the anterior cingulate cortex is a component of the limbic loop and is deeply associated with anticipation of negative events (Straube et al., 2009). Therefore, while the PeF has not been identified as a dysfunctional brain region in OCD, its downstream regions within the CSTC loops may contribute to the manifestation of phenotypes observed in OCD.

The security motivation system, proposed by Szechtman and Woody, offers a notable model for understanding the pathophysiology of OCD, which closely aligns with our present findings (Szechtman and Woody, 2006; Woody and Szechtman, 2011). According to this model, the security motivation system can be considered as an independent module in the brain that has evolved in response to adaptive concerns regarding potentially catastrophic risks, such as predation and diseases (Woody and Szechtman, 2011). This system encompasses a set of biologically primitive behaviors, including checking and washing, which are triggered by perceived potential threats (Szechtman and Woody, 2006; Woody and Szechtman, 2011). While individuals without OCD possess a functional security motivation system that enables them to evaluate and cope with potential threats appropriately, dysfunction in this system can result in the overestimation of potential threats and the manifestation of repetitive and excessive motor actions, such as compulsive checking and washing (Woody and Szechtman, 2011). Although brain regions composing the security motivation system have not been definitively proven, our study supports this theoretical model, and the hypothalamus is a strong candidate to drive this system (Figure 5A). Our study suggests the involvement of hypothalamic neurons and their downstream circuits in the modulation of home security, anticipation of potential threats, and repetitive/stereotypic behaviors. Therefore, compulsive behaviors observed in OCD may be induced by defensive instincts or safety needs. Further research is necessary to validate and expand upon these findings, which nevertheless offer valuable insights into the neural mechanisms underlying security-related behaviors and their potential therapeutic implications.

Loss-of-function approaches are the most straightforward methods to demonstrate the indispensable role of PeF UCN3 neurons in the induction of ES-like behavior. For example, to investigate the role of these neurons in ES-like behavior, their selective ablation is conducted by forced expression of diphtheria toxin. However, such experiments pose challenges because physiological or external stimuli and housing conditions that reliably induce ES-like behavior remain unclear. Thus, it is almost impossible to assess how the ablation of these neurons impacts ES-like behavior. Although lactation is a physiological state that positively influences the ES-like behavior (Calhoun, 1963), its occurrence rate and reliability are too low to obtain a sufficient sample size for statistical analysis of the behavior. In the future, establishing a new paradigm of the induction of ES-like behavior with high probability, such as the resident-intruder paradigm for aggression, would promote our understanding of the neural mechanisms underlying the anticipation of risks and the security system. Cell populations and regions activated by CNO administration were identified through a histological approach with the neuronal activity marker c-Fos. Therefore, it is necessary to assess their functional responses through different approaches, such as whole-cell patch clamp recording. Our histological experiments and previous research investigating the relevance of the LS for burying behavior suggest the involvement of LS neurons in both burying and ES-like behaviors. However, selective activation of the neural pathway between PeF UCN3 neurons and the LS using the optogenetic approach is necessary to confirm the relevance of LS neurons for ES-like behavior. Furthermore, our findings are limited to the mouse model, and further studies, including translational research, are important to understand the pathophysiology of OCD and develop anti-OCD drugs with a new mechanism of action.

## Data availability statement

The original contributions presented in this study are included in this article/Supplementary material, further inquiries can be directed to the corresponding author.

## Ethics statement

The animal study was approved by the Animal Care Committee of Nara Medical University. The study was conducted in accordance with the local legislation and institutional requirements.

## Author contributions

NH-H: Conceptualization, Data curation, Formal analysis, Funding acquisition, Investigation, Methodology, Project administration, Validation, Writing—original draft. KM: Data curation, Formal analysis, Investigation, Writing—review and editing. TK: Investigation, Writing—review and editing. KK: Resources, Writing—review and editing. AI: Resources, Writing—review and editing. MFN: Conceptualization, Writing—review and editing. KT: Conceptualization, Writing—review and editing. KI: Supervision, Writing—review and editing. MNi: Funding acquisition, Supervision, Writing—review and editing.
