# Supplementary material for: Entrance-sealing behavior in the home cage: a defensive response to potential threats linked to the serotonergic system and manifestation of repetitive/stereotypic behavior in mice
Source: Front Behav Neurosci. 2024 Jan 5;17:1289520. doi: 10.3389/fnbeh.2023.1289520 (PMC10799337; doi:10.3389/fnbeh.2023.1289520)
Supplement: Supplementary file 1 [file Data_Sheet_1.PDF]

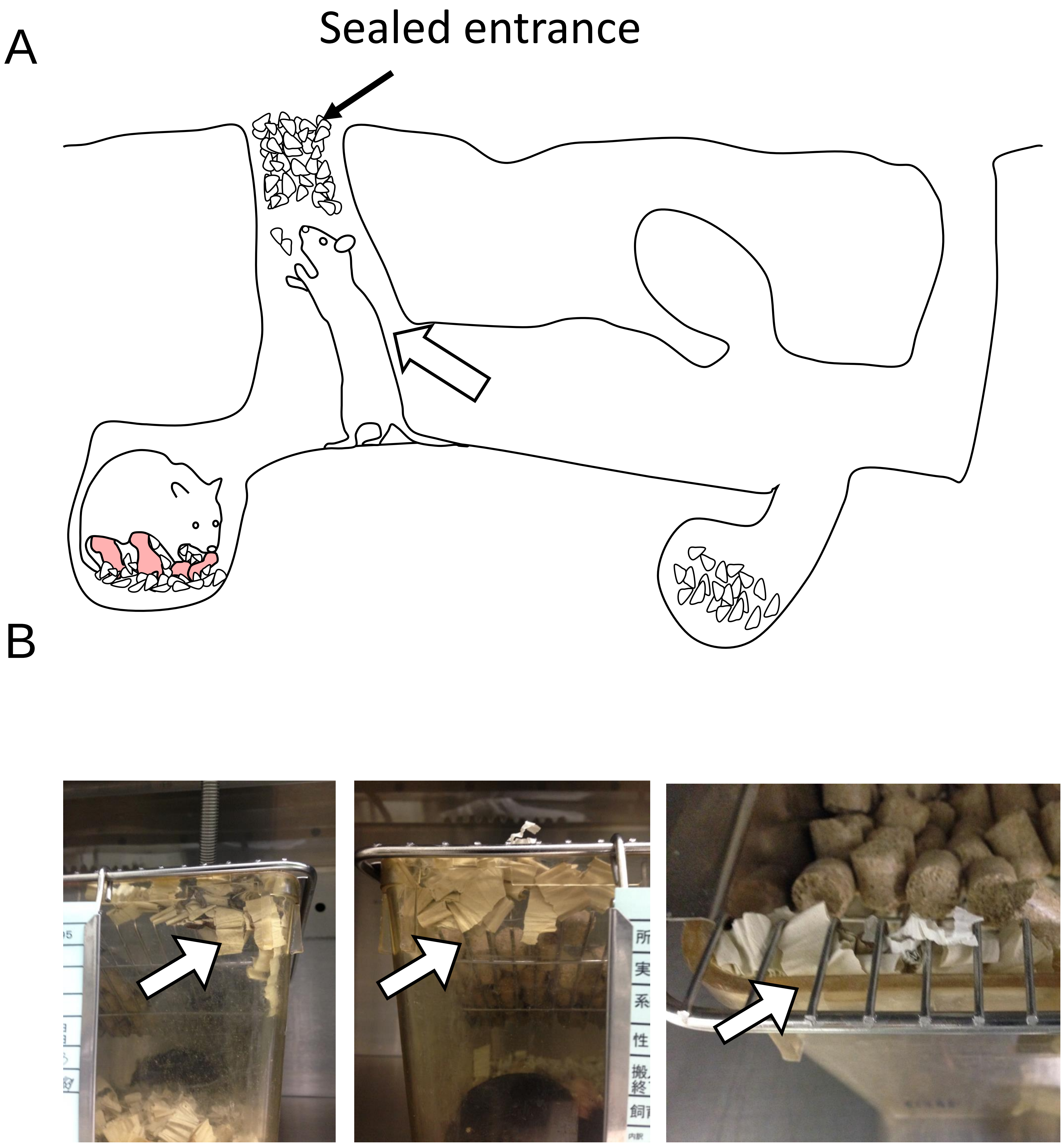


**Figure S1** ES behavior in wild rats and examples of ES-like behavior in laboratory mice **A.** Illustration showing ES behavior in wild rats. Rats seal entrances to their burrows from the inside using materials such as mud, sand, and vegetation to reinforce the security of their habitats. Various factors, including lactation/the presence of pups, contribute to the promotion of ES behavior. **B.** Photographs displaying traces of ES-like behavior observed in laboratory mice, specifically lactating females. Each photograph shows the traces of ES-like behavior, which includes the openings of the cage lid sealed with bedding material and feces.

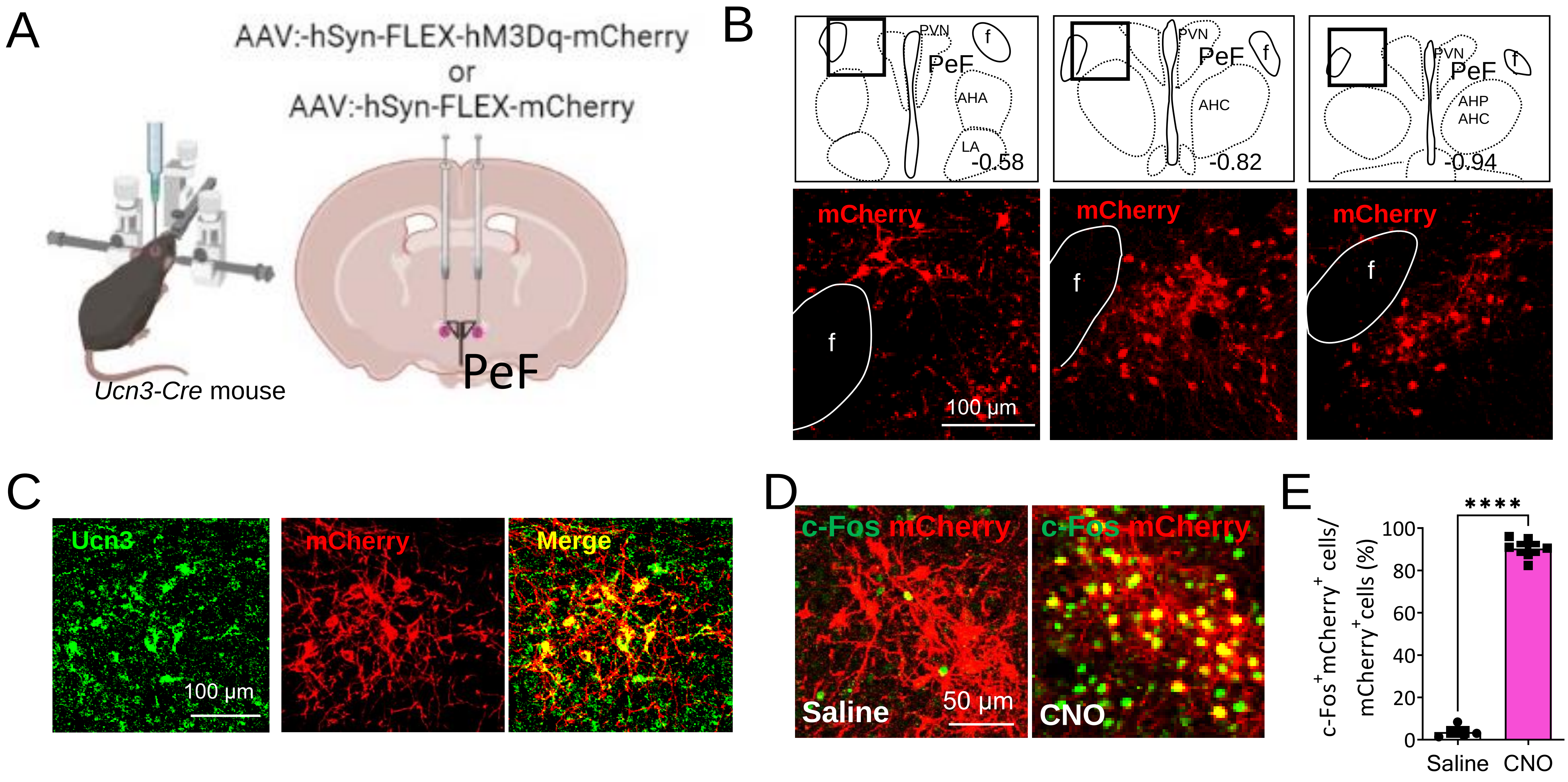


**Figure S2** Chemogenetic activation of PeF UCN3 neurons using Ucn3-Cre mice **A.** Schematic diagram illustrating the targeted stereotaxic injection of AAV vectors into the PeF of Ucn3-Cre mice. These vectors are capable of inducing Cre-dependent expression of hM3Dq-mCherry or mCherry specifically within the PeF. **B.** Fluorescent images showing the distribution of mCherry-labeled cells in the PeF at different anterior-posterior coordinates relative to the bregma line. The images represent sections at 0.58 mm (left), 0.82 mm (middle), and 0.94 mm (right) posterior to the bregma. The squared regions on the map indicate the field of microscopic view beneath, providing a focused view of the specific area of the interest in the PeF. **C.** Fluorescent images demonstrating the co-localization of Ucn3-labeled cells (green) and mCherry-labeled cells (red) in the PeF. **D.** Fluorescent images showing c-Fos expression (green) in mCherry-labeled cells after saline (left) or CNO (right) injection. **E.** Graph illustrating the percentage of c-Fos and mCherry double-labeled cells to the total number of mCherry-labeled cells (n=5, 5, unpaired t test. ****$P<0.0001$, saline: 2.12±0.47%, CNO: 96.0±0.34%).

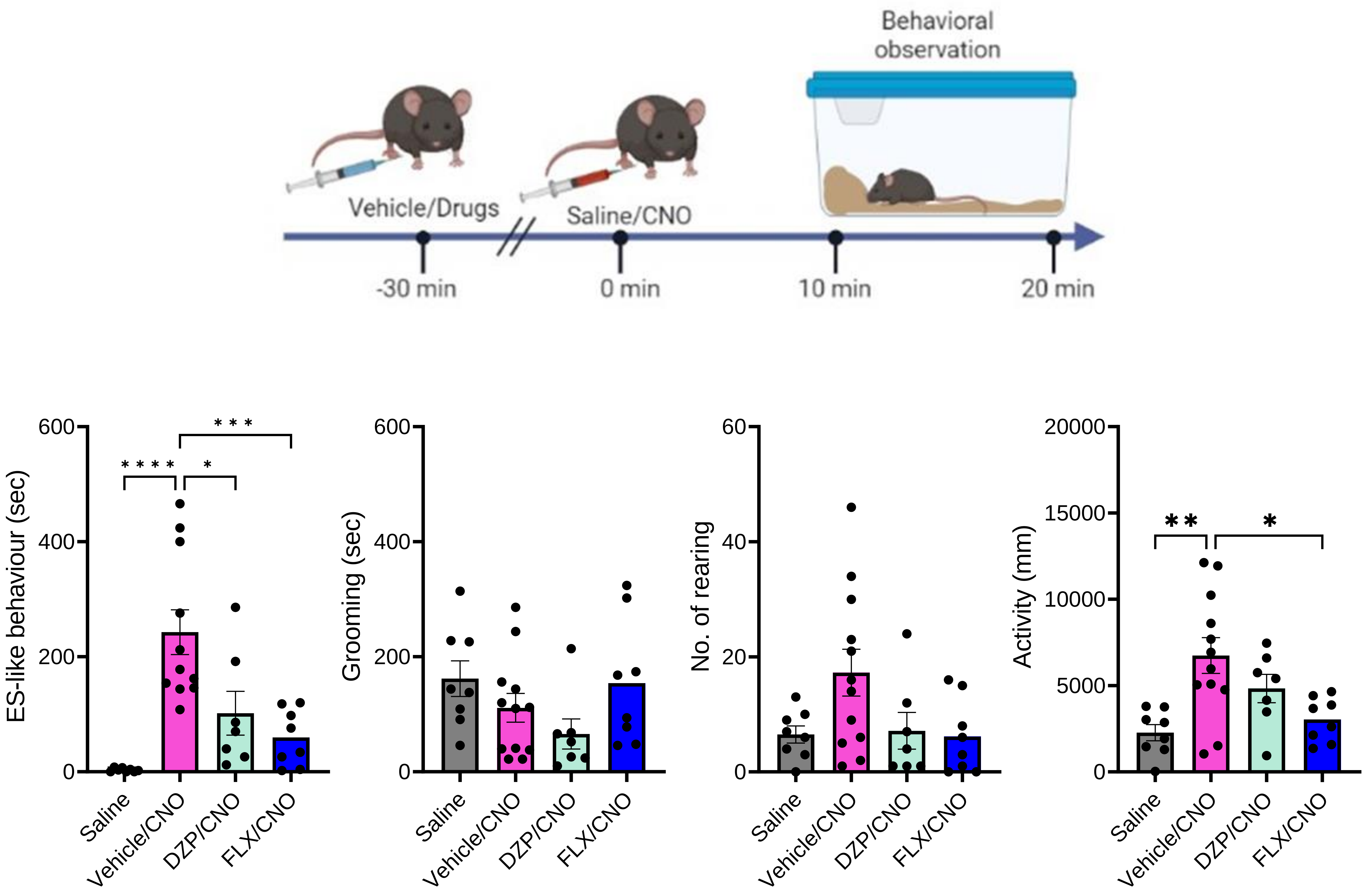


**Figure S3** The effects of DZP and FLX on home-cage behaviors. **A.** Timeline of behavioral observation in mice after injection with vehicle or drugs, followed by CNO administration (Time -30 min: injection of vehicle or drugs, Time 0 min: CNO administration, Time 10–20 min: behavioral observation within the home-cage). **B.** Graphs showing the duration of ES-like behavior, the number of rearing, and activity (n=8–11, unpaired one-way ANOVA followed by Turkey's post-hoc test) and the duration of grooming (n = 8-11, Kruskal–Wallis's test followed by Dunnett's post-hoc test. Post-hoc results are indicated in the graphs). *P<0.05, **P<0.01, ****P<0.0001.

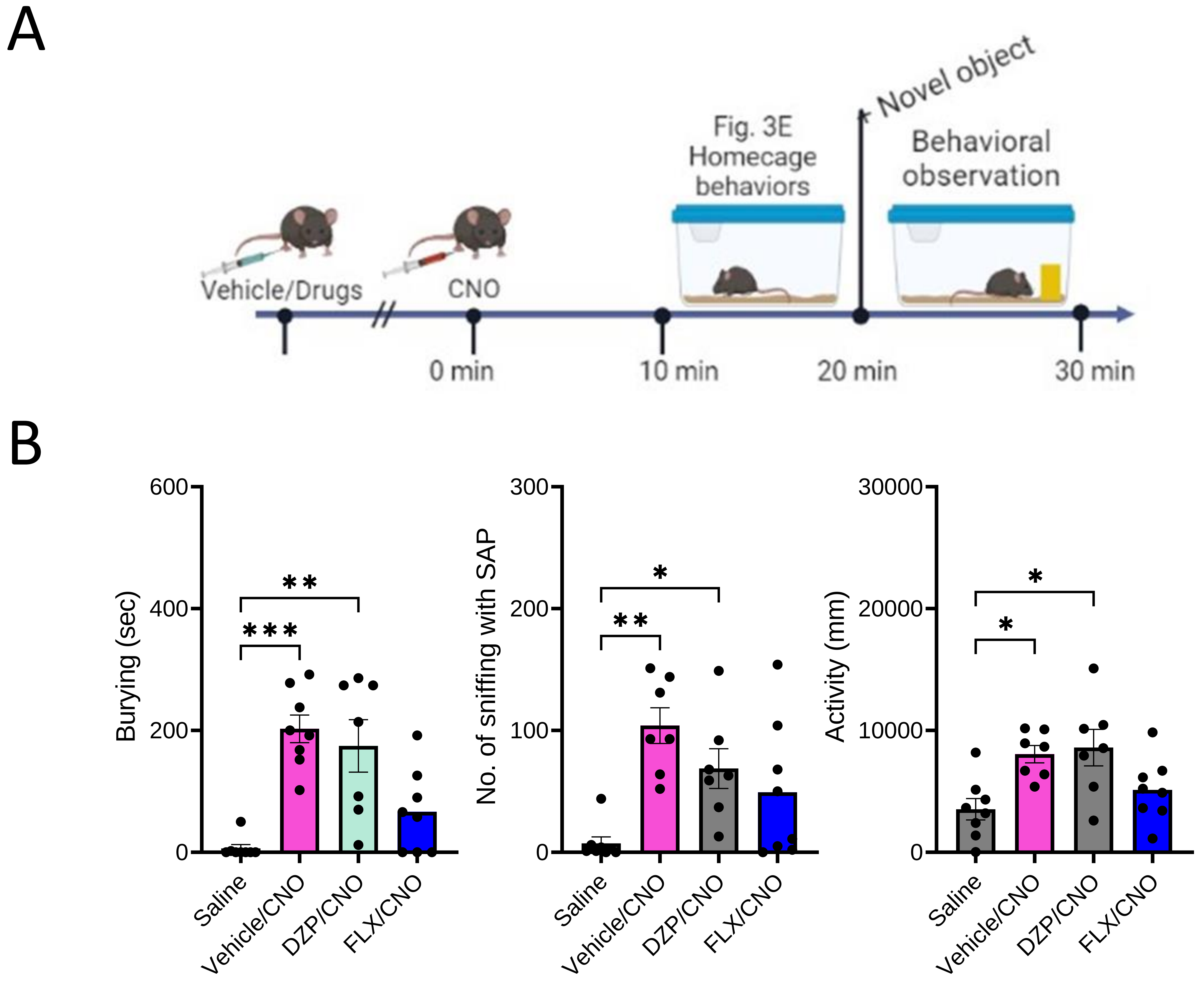


**Figure S4** The effects of DZP and FLX on burying behavior **A.** Timeline of object-burying test in the home-cage after injection with vehicle or drugs, followed by CNO administration (Time -30 min: injection of vehicle or drugs, Time 0 min: saline/CNO administration, Time 10–20: Observation of home-cage behavior, Time 20–30 min: observation of behavioral responses to a novel object). **B.** Graphs showing the duration of burying, number of sniffing with SAP, and activity in mice after injection of vehicle, DZP, or FLX, followed by CNO administration (n=7–8, Kruskal–Wallis followed by Dunnett's post-hoc test; post-hoc results are indicated in the graphs, *** $P<0.001$, ** $P<0.01$, * $P<0.05$; ANOVA results: burying, *** $P<0.0001$, the number of sniffing with SAP, ** $P<0.01$, ** $P<0.01$, activity, ** $P<0.01$)

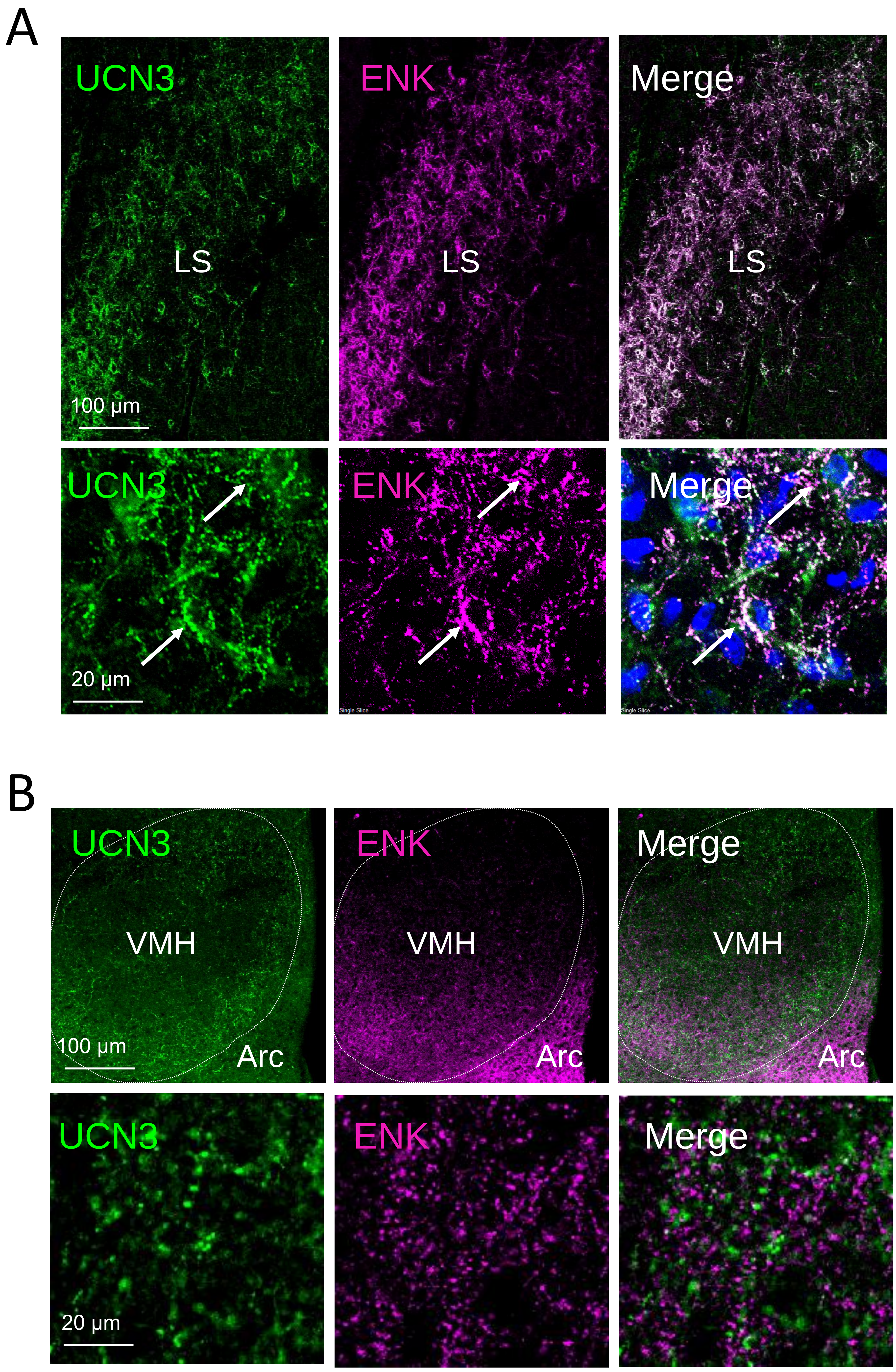


**Figure S5** The colocalization of UCN3-labeled and ENK-labeled nerve fibers surrounding LS neurons **A.** Lower (top) and higher (bottom) resolution images showing the colocalization of UCN3-labeled (green) and ENK-labeled (magenta) nerve fibers in the LS. Arrows indicate UCN3/ENK labeled perisomatic baskets around LS neurons (blue: DAPI). **B.** Lower (top) and higher (bottom) resolution images demonstrating double labeling of UCN3 (green) and ENK (magenta) in the VMH.
